# CD4:CD8 ratio in children with perinatally acquired HIV‐1 infection†

**DOI:** 10.1111/hiv.12642

**Published:** 2018-08-06

**Authors:** T Seers, P Vassallo, K Pollock, JP Thornhill, S Fidler, C Foster

**Affiliations:** ^1^ Faculty of Medicine Imperial College London London UK; ^2^ Imperial College Healthcare NHS Trust London UK

**Keywords:** CD4:CD8 ratio, perinatally acquired HIV infection

## Abstract

**Objectives:**

In adults with horizontally acquired HIV infection, an inverted CD4:CD8 ratio is associated with persistent immune activation, size of HIV reservoir and predicts an increased risk of non‐AIDS‐defining adverse events. Normalization of this ratio with antiretroviral therapy (ART) is suboptimal in adults, despite viral suppression, and is less well described in paediatric populations. We investigated rates of CD4:CD8 ratio recovery in children with perinatally acquired HIV infection (PaHIV) on ART.

**Methods:**

A cross‐sectional, retrospective analysis of routine clinical data in children with PaHIV (5–18 years old) attending a single UK centre was carried out.

**Results:**

CD4:CD8 normalization was seen in 62% of children on suppressive ART. A negative correlation was found between current CD4:CD8 ratio and age at start of ART. Positive correlations were found between current CD4:CD8 ratio and total time with suppressed HIV viral load and nadir CD4 counts. Multiple linear regression analysis showed that age at start of ART was significantly associated with current CD4:CD8 ratio (standardized β = −0.680; *P* < 0.001). Patient sex, ethnicity and antiretroviral regimen did not affect ratio recovery.

**Conclusions:**

We found higher rates of CD4:CD8 ratio normalization compared with previous adult studies. Children who started ART at a younger age were more likely to recover a normal ratio. The current policy of universal treatment for all HIV‐positive adults and children will enhance immunological normalization.

## Introduction

There are currently 1.8 million children under 15 years of age living with HIV infection worldwide, of whom 95% have perinatally acquired HIV infection (PaHIV), with treatment recommended for all irrespective of age and immunological status 1, 2. As the proportion of HIV‐positive children on antiretroviral therapy (ART) increases annually 2, there has been a shift towards seeking a greater understanding of the long‐term clinical impact of well‐controlled chronic HIV infection in children. Data in adults suggest that even very well‐controlled infection may be associated with an increased risk of non‐AIDS‐defining adverse events 3.

An inverted CD4:CD8 ratio (< 1) may be a biomarker for underlying immune activation and increased risk of non‐AIDS‐defining adverse events 3, 4, and can be derived from routinely measured clinical data. A low CD4:CD8 ratio (< 1) may reflect ongoing subclinical inflammation, driving an increased CD8 T‐cell frequency, and it is associated with an immune senescent immunophenotype both in older non‐HIV‐infected adults and in adults living with HIV 4. In one small PaHIV study, an inverted CD4:CD8 ratio was associated with markers of increased immune activation and senescence, despite plasma virological suppression with ART 5. In addition, levels of inflammatory markers (e.g. interleukin‐6) pre‐ART are associated with end‐stage events [death or World Health Organization (WHO) clinical stage 4] independent of CD4 count 6. Surrogate markers for pre‐existing and ongoing inflammation may therefore play an important future role in evaluating treatment success and predicting both AIDS‐defining and non‐AIDS‐defining outcomes. This is supported by adult studies showing that traditional markers of disease progression (e.g. CD4 count) alone are unable to predict the risk of non‐AIDS‐defining adverse events 7.

Worryingly, the association between increased risk of cardiovascular events and low CD4:CD8 ratio was strongest in younger adults < 50 years old 8 and may have implications for children and young adults living with HIV, who may already display surrogate markers for increased risk of cardiovascular disease 9.

In adults where ART was started in chronic infection, CD4:CD8 ratio normalization is uncommon, with one large study showing less than one‐third of patients achieving a normal ratio despite 5 years of suppressive ART 10. Other studies have shown that ART initiation closer to seroconversion is associated with increased numbers of individuals with normalization of the CD4:CD8 ratio 11. ART regimen may also affect the likelihood of CD4:CD8 normalization in adults 12. However, these associations are much less well defined in children with PaHIV, who are arguably most at risk of non‐AIDS‐defining morbidity given the chronicity of infection with HIV. This makes interventions to modify any potential risk an urgent clinical need. In PaHIV, the time of infection usually corresponds to birth, making this a unique opportunity to explore the impact of length of infection prior to ART initiation. In this study, we examined the relationship between CD4:CD8 ratio and parameters of ART timing, regimen and immunological response in children with PaHIV.

## Methods

Routinely collected clinical data were extracted from the records of patients from the paediatric HIV service at a UK university teaching hospital. Patients were all perinatally infected with HIV and aged between 5 and 18 years at the time of inclusion in the study. CD4:CD8 ratios were measured by standard flow cytometry. Current virological suppression was defined as HIV viral load (VL) < 50 HIV‐1 RNA copies/mL. Time suppressed on ART was calculated from the first recorded date at which the VL was < 50 copies/mL. Any single measurement where the VL was > 50 and < 400 copies/mL was considered a viral ‘blip’. Any measurement of VL > 400 copies/mL was considered to be a failure of virological suppression. The age at start of ART was calculated from paper notes, when recorded. First‐line ART was defined as initiation of triple therapy consisting of two nucleoside reverse transcriptase inhibitors and a boosted protease or nonnucleoside reverse transcriptase inhibitor, with second‐line therapy defined as a change in at least one drug class following virological failure. Data were anonymized and then Spearman's rho was calculated and the Mann–Whitney *U*‐test and multiple linear regressions were performed in spss statistics V24.0 (Armonk, NY, USA), with a *P*‐value of < 0.05 considered significant. Data were analysed following Health Research Authority guidance on the use of routinely acquired clinical data for research. A CD4:CD8 ratio of ≥ 1 was considered normal 5.

## Results

### Patient characteristics and successful CD4:CD8 ratio normalization

A total of 112 perinatally infected individuals, aged 5–18 years, were included in this study. Of these, 105 of 112 (94%) were on ART at the time of the study, and 93 of 112 (83%) had an undetectable VL (< 50 copies/mL) at the time of CD4:CD8 ratio measurement. The demographic characteristics of the cohort at the time of CD4:CD8 ratio measurement are shown in Table 1.

**Table 1:** Cohort characteristics (*n* = 112)

Current age (years) [median (IQR)]	15.4 (12.6–16.8)
Sex [*n* (%)]	
Female	65 (58)
Male	47 (42)
Ethnicity [*n* (%)]	
Black African	86 (76.8)
Black other	13 (11.6)
Caucasian	6 (5.4)
Caucasian, South East Asian	4 (3.6)
South Asian	3 (2.7)
Current ART therapy [*n* (%)]	
On ART	105 (93.8)
Not on ART	7 (6.3)
First‐line ART [*n* (%)]	48 (51.6)
Other ART [*n* (%)]	45 (48.4)
Age (months) at start of ART [median (IQR)] (*n* = 93)	39.8 (5.6–103.4)
Length of follow‐up from start of ART (months) [median (IQR)] (*n* = 89)	128 (59–161)
CD4:CD8 ratio ≤ 0.4 [*n* (%)]	11 (9.8)

ART, antiretroviral therapy; IQR, interquartile range.

In the 93 patients on suppressive ART at the time of CD4:CD8 ratio measurement, the median CD4:CD8 ratio was 1.1 [interquartile range (IQR) 0.8–1.6; range 0.3–2.7] (Table 2). Fifty‐eight of 93 (62%) patients had a normal CD4:CD8 of ≥ 1 and 35 (38%) had a ratio of < 1. When comparing those with a normal CD4:CD8 ratio and those with a ratio < 1, there was no significant difference in sex or ethnicity, although the majority were black African. There was no significant difference in the median CD4:CD8 ratio at the start of ART between those with a ratio ≥ 1 and those with a ratio of < 1 at study close. Very low CD4:CD8 ratios (≤ 0.4) have been associated with increased rates of non‐AIDS‐defining adverse events 10. Eleven patients were in this group, with five of these having an undetectable VL at study close.

**Table 2:** Relative characteristics of patients with a CD4:CD8 ratio ≥ 1 and < 1, for patients with a viral load < 50 copies/mL (*n* = 93)

	CD4:CD8 < 1 (*n* = 35)	CD4:CD8 ≥ 1 (*n* = 58)	*P*‐value
Age (years) [median (IQR)]	16.3 (14.9–16.7)	14.8 (12.1–16.8)	0.03
Sex female [*n* (%)]	19 (54)	35 (60)	0.56
Ethnicity black African [*n* (%)]	27 (77)	45 (78)	0.96
Absolute CD4 count (cells/μL) [median (IQR)]	667 (528–784)	1033 (704–1373)	< 0.01
Absolute CD8 count (cells/μL) [median (IQR)]	959 (841–1146)	703 (510–850)	< 0.01
Nadir CD4 count (cells/μL) [median (IQR)]	260 (170.5–500.5)	480 (283–685)	< 0.01
Time suppressed on ART (months) [median (IQR)]	57 (22–107)	112 (62.3–139.3)	0.002
Age at start of ART (months) [median (IQR)] (*n* = 88)	113.4 (63.7–148)	21.1 (4.1–45.8)	< 0.01
CD4:CD8 at start of ART [median (IQR)] (*n* = 57)	0.38 (0.19–0.64)	0.61 (0.35–1.19)	0.06

### CD4:CD8 ratio and ART initiation and duration

The age at start of ART could be determined in 88 cases. A strong negative correlation between age at ART start and current CD4:CD8 ratio (rho = −0.577; *P* < 0.01) was observed in this group. The median CD4 count at the start of ART was 460 cells/μL (IQR 279–950 cells/μL), and counts were available for 81 individuals. There was a positive correlation between the total length of time suppressed on ART and current CD4:CD8 ratio (rho = 0.418; < 0.01).

There was no significant difference in the proportion of patients with normal CD4:CD8 ratios between patients on first‐line ART and those on second‐line or subsequent therapy at the time of CD4:CD8 measurement. There was a positive correlation between nadir CD4 count and current CD4:CD8 ratio (rho = 0.408; *P* < 0.01).

### Determinants of CD4:CD8 ratio recovery

Of the 41 patients with a CD4:CD8 ratio < 1 at start of ART, 14 did not normalize their ratio following ART. There was no significant difference in the length of time suppressed on ART between those who were able to subsequently normalize their ratio and those with a persistently inverted ratio (*P* = 0.229). However, in the 55 cases in which age at start of ART was also known, there was a significant difference in the age at start of ART between the two groups [CD4:CD8 < 1, median 97.2 months (IQR 18.5–120.5 months) *vs*. CD4:CD8 ≥ 1, median 13.0 months (IQR 3.9–44.1 months); *P* = 0.008].

Multiple linear regression analyses were performed to determine factors associated with CD4:CD8 ratio recovery. CD4:CD8 ratio was associated with age at start of ART (standardized β −0.680; *P* < 0.001). However, there was no association with sex (β = −0.118; *P* = 0.222), VL at the start of ART (β = 0.177; *P* = 0.86) or nadir CD4 count (β = 0.172; *P* = 0.138). Total time on ART was collinear with age at start of ART and therefore was not included in the model. The fit of the model (*r* ^2^) was −0.383.

## Discussion

In this study, perinatally infected children initiated ART at a median of 3 years of age, in the chronic stage of HIV infection, with a median of 10.7 years of follow‐up. However, with suppressive ART, almost two‐thirds achieved a normal CD4:CD8 ratio at the time of analysis. This is a significantly higher proportion than has been reported in adults starting ART in chronic infection 10. However, adults starting ART in early HIV infection appeared to have a similarly enhanced capacity for ratio normalization 11, and while this was not as marked as in the PaHIV individuals in our cohort, this PaHIV cohort was followed up for over twice as long 11.

A persistently abnormal CD4:CD8 ratio was more likely in children who were older and who started ART at an older age. This finding may have been confounded by the cohort; among PaHIV children currently in the UK, the older children are likely to have started ART at a more advanced stage of infection 1.

Adult studies have shown that normalized CD4:CD8 ratio is predicted by higher baseline CD4 counts, lower CD8 counts, longer length of time with a suppressed VL, younger age and higher CD4:CD8 at ART start 8, 10. This study replicates these findings in perinatally infected children, while highlighting that a significantly greater proportion of children have a normal CD4:CD8 ratio when suppressed on ART compared with adults. In addition, we observed a strong positive correlation between nadir CD4 count and current CD4:CD8 ratio, which has not been reported in previous smaller studies in children 5.

While different study designs and different definitions of a ‘normal’ CD4:CD8 ratio make direct comparisons difficult, in two adult studies in which the length of time on ART was comparable to that in this study, there were still considerably lower rates of ratio normalization (37% and 16%, respectively)^13^, ^14^. However, nadir CD4 counts were lower in both adult cohorts (medians 130 cells/μL and 157 and 136 cells/μL for the CD4:CD8 ≥ 0.9 and < 0.9 groups, respectively) compared with this cohort (median 425 cells/μL).

Multiple differences between the paediatric and adult immune responses to HIV may contribute to the difference in the ability to normalize CD4:CD8. It is thought that a selective expansion of T regulatory lymphocytes in paediatric HIV infection, which is not present in chronic HIV infection in adults, could play a role in regulating ongoing immune dysfunction/activation 15. Additionally, the proliferative capacity of HIV‐specific CD4 cells can be improved in children on ART, a capacity lost in chronically infected adults 15. More broadly, the tolerogenic bias towards T helper type 2 (Th2), and away from Th1, in infancy may limit immune activation in children with HIV infection 16. These variations may explain the increased capacity for ratio normalization observed in our paediatric cohort.

Similarly to adult studies, we found that the length of time living with HIV, but not on ART, also predicted ratio recovery 11. Adults starting ART at < 6 months from seroconversion had much higher rates of CD4:CD8 recovery at 1 year (46.9%) than those starting at ≥ 6 months (12.6%) 11. Although this could explain some of the improved ratio recovery in this cohort, the median age at start of ART for children with a current CD4:CD8 ≥ 1 was still significantly greater than 6 months (median 21 months). This difference further supports the enhanced capacity for immunological recovery in paediatric cohorts not seen among adults.

Children who started ART at a younger age had a higher CD4:CD8 ratio. We also found a subgroup of children who on average started ART when older and, despite being on suppressive ART for comparable amounts of time, were not able to normalize CD4:CD8. This could be explained by the association between earlier ART and greater reductions in viral reservoir size, seen in both paediatric 17 and adult 18 cohorts, with this effect persisting for longer in children 19. Additionally, viral replication drives persistently elevated CD8 counts 20 and is closely related to ongoing inflammation 16.

This study had a number of limitations. Despite our centre being one of the largest in our region, we were only able to include 112 patients. Additionally, we were not able to record either time to viral suppression or data on historical treatment regimen – both factors may influence the CD4:CD8 ratio in adults 10. Importantly, the definition of a ‘normal’ CD4:CD8 ratio in children living with HIV remains unclear. While a ratio of < 1 is clearly abnormal and characteristic of childhood HIV infection, it is less clear what a healthy ratio might be. Published reference ranges for uninfected children suggest an optimal ratio > 1, highlighting the need for further research to maximize the utility of the CD4:CD8 ratio as a marker of treatment response 21.

The link between a persistently abnormal CD4:CD8 ratio and worse outcomes in adults living with HIV is complex 7, 22. Any increase in mortality is likely to be secondary to persistent immune dysregulation or an immunosenescent phenotype 4, 22. Therefore, as long‐term survival for children with HIV infection becomes the norm and the adolescent HIV‐infected population grows, it will be increasingly important to identify individuals at highest risk, despite normalized CD4 counts, and ensure that interventions to limit risk are in place. The relationship between early ART and normalized CD4:CD8 ratio described here supports the current strategy of early diagnosis and universal treatment for all children living with HIV.
